# Temperature Effect on Stacking Fault Energy and Deformation Mechanisms in Titanium and Titanium-aluminium Alloy

**DOI:** 10.1038/s41598-020-60013-6

**Published:** 2020-02-20

**Authors:** Beikai Zhao, Peng Huang, Libo Zhang, Suzhi Li, Ze Zhang, Qian Yu

**Affiliations:** 1 Center of Electron Microscopy and State Key Laboratory of Silicon Materials, School of Materials Science and Engineering, Zhejiang University, Hangzhou, 310027 China; 2 State Key Laboratory for Mechanical Behavior of Materials, Xi’an Jiaotong University, Xi’an, 710049 China

**Keywords:** Materials science, Structural materials

## Abstract

Alloying elements have great influence on mechanical properties of metals. Combining dislocation characterization and *in-situ* transmission electron microscope straining at ambient and liquid-nitrogen temperature in high-purity titanium and Ti-5at%Al, we investigated the modulation of Al on dislocation behaviours as temperature changed. It reveals that segregation of Al at edge dislocation cores in Ti-5at%Al generates strong obstacles, promoting room temperature cross-slips. However, the effect of Al on reducing stacking-fault energy (SFE) as decreasing temperature is significant. Consequently, the lower SFE in Ti-5at%Al results in ordinary planar dislocation slip while massive dislocation cross-slips occurred in Ti at liquid-nitrogen temperature.

## Introduction

The mechanical properties of materials are highly related to defect behaviours, which both are practically modulated by materials processing and treatments. Strength is determined by how difficult the dislocations can move while ductility contrarily requires easy motion of dislocations^1,2^. Alloying is one of the most important means to optimize metal materials through adding other elements into origin materials to obtain desired properties which adapt to the actual applications^3–5^. Alloying as the nuclear technique of metal materials preparation and upgrading craft is mainly via the solid solution strengthening mechanism which improve strength or impart ductility deriving from the misfit of the interstitial or substitution atoms with solution atoms and their interaction with defects such as dislocations^6,7^. Alloying elements can distribute randomly, forming random solid solutions; each solute atom generates an individual stress-strain field influencing dislocation movements in crystals like hindering dislocation glide^8,9^. Besides, alloying elements can also reunion at some places generating segregations. Segregation distributes dispersive in matrix forming Cottrell atmosphere pinning dislocations while segregations atoms can also gather in defects like dislocation cores, leading to non-linear elastic interaction^10–12^.

On the other hand, the addition of alloying elements does effects on the stacking fault energy (SFE)^13–15^. It is known that SFE affects the priority of different deformation mechanisms, therefore make changes on mechanical properties^13,16,17^. Generally, low-SFE materials are more likely to deform by partial dislocations or twinning while high-SFE promotes full dislocation motion and cross slip. The addition of alloying elements modulates SFE since solute atoms may segregate in stacking fault plane between partial dislocations of extended dislocation. Hence, as content of solute atoms increases, two partial dislocations separate further leading the extended dislocation is hard to move.

Importantly, the effect of alloying elements and the influence it does on dislocation movements are adjusted by temperature which would consequently result in significant change on mechanical performance of materials^14^. In details, Peirls-Nabarro force that theoretically used to describe the stress for activating dislocation glide is highly related to the value of lattice friction stress. However, since lattice vibration does always exist above 0 K and changes with temperature, it influences the value of lattice friction, the local variation of lattice friction and the energy barriers for dislocation glide. In addition, SFE is also a function of temperature. It was demonstrated that density of vacancy varies with temperature, which can influence the elastic strain energy among lattices in alloys^18^. Hence, the energy that stacking faults need to change structure is influenced. Generally speaking, dislocation behaviours like dislocation climb and dislocation cross-slip are easier to occur at high temperature; as temperature decreases, lattice friction will increase correspondingly causing critical resolved shear stress (CRSS) increase, promoting deformation twinning^13,19,20^. Nevertheless, since the practical materials are often used in different temperature environment, understanding the effect of temperature on the alloying effect on deformation mechanisms not only help us understand the solid solution strengthening mechanism at different temperature but also give us direction of actual applications of alloy materials.

Ti is generally used as significant materials such as airframes, aeroengines due to its high strength, high corrosion resistance, and special high strength-weight ratio. Comparing with other metals or alloys, titanium keeps excellent properties in extreme environment like seawater desalination which need to serve at low temperature^20,21^. Understating the change of deformation mechanisms in Ti materials and how alloying element affect the defect behaviours as decreasing temperature is of interest^22–24^. In this study, the deformation mechanisms of high-purity Ti and Ti solid solution with 5% atomic ratio of Al (Ti-5at%Al) are studied at both room temperature and liquid-nitrogen temperature, respectively, through atomic resolution crystal structure and chemical structure characterization, *in-situ* TEM deformation and atomistic simulations. By comparing the mechanical deformation of two materials, we clearly reveal the influence of temperature on the alloying effect and the corresponding deformation mechanisms.

The TEM samples of single crystal high-purity Ti and single crystal Ti-5at%Al were prepared using twin-jet polishing and through high angle annular dark field scanning transmission electron microscope with energy dispersive X-ray spectroscopy (EDS) and electron energy loss spectroscopy (EELS), we characterised the crystal and chemical arrangements of Ti-5at%Al at dislocation cores. By using tensile holder, *in-situ* straining tests were performed inside TEM and relevant details were described in previous paper^25^. The *in situ* tensile tests were performed along different directions in Ti and Ti-5at%Al, respectively, to make comparison. In each comparison, the zone axis and the pulling direction were the same in Ti and Ti-5Al.

## Results and Discussion

**Figure 1:**
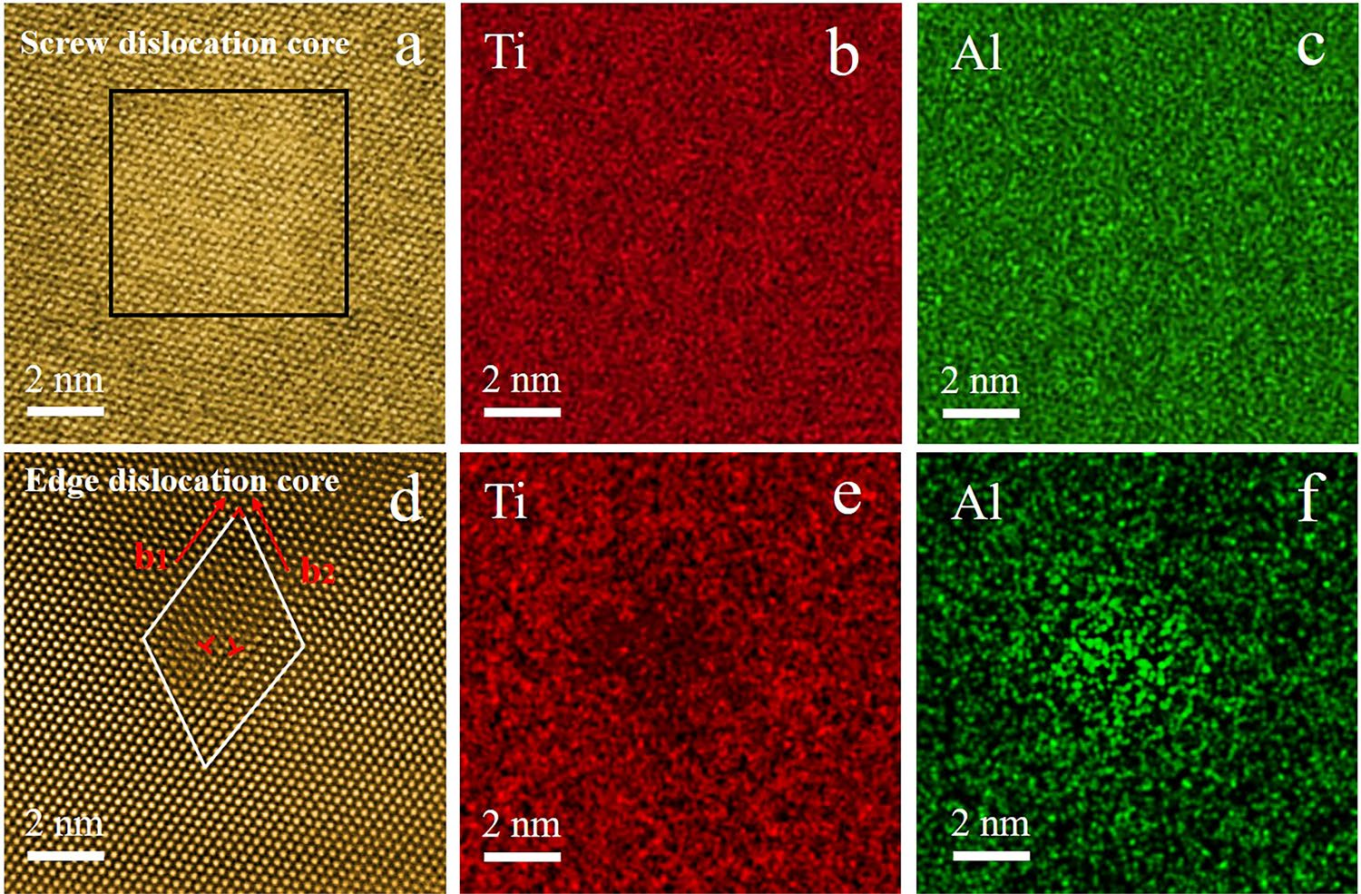
(**a**) HAADF-STEM image of the screw dislocation core and EDS scanning results of (**b**) Ti element distribution and (**c**) Al element distribution; (**d**) HAADF-STEM image of the edge dislocation core with two half atom surface (${b}_{1}=1/3[\bar{2}110]$, ${b}_{2}=1/3[\bar{2}113]$) and EDS scanning results of (**e**) Ti element distribution and (**f**) Al element distribution. All the beam direction is $[11\bar{2}0]$.

It has been broadly reported that even a small amount of alloying atoms (several atomic percentage or even point several atomic percentage) can make significant impact on materials mechanical properties^11^. The alloying effect on the mechanical properties is certainly related to the type of solute atoms, while the next coming question is where the alloying atoms go. We characterized the crystalline and chemical distribution of atoms in the matrix of Ti-5at%Al alloy, at the edge dislocation core and screw dislocation core, respectively. It was observed that the Ti and Al atoms distribution in solution and at the screw dislocation core was quite uniform while Al atoms showed tendency to segregate at edge dislocation core. The atomic resolution images of screw dislocation core were obtained by tracking a screw dislocation through tilting. The screw dislocation core was displayed in the area of rectangle in Fig. 1(a) while the Fig. 1(b,c) showed the elements distribution of this district. The Fig. 1(b,c) illustrated although adding the aluminium to the titanium, the distribution of two elements were distributed evenly without segregation of solute atoms. Figure 1(d) shows an edge dislocation core in Ti-5at%Al of two edge dislocations with Burgers vector ${b}_{1}=1/3[\bar{2}110]$ and ${b}_{2}=1/3[\bar{2}113]$. The corresponding EDS results revealed that Al atoms segregate at the edge dislocation core as shown in Fig. 1(e,f), which is different from the situation around the screw dislocation core.

**Figure 2:**
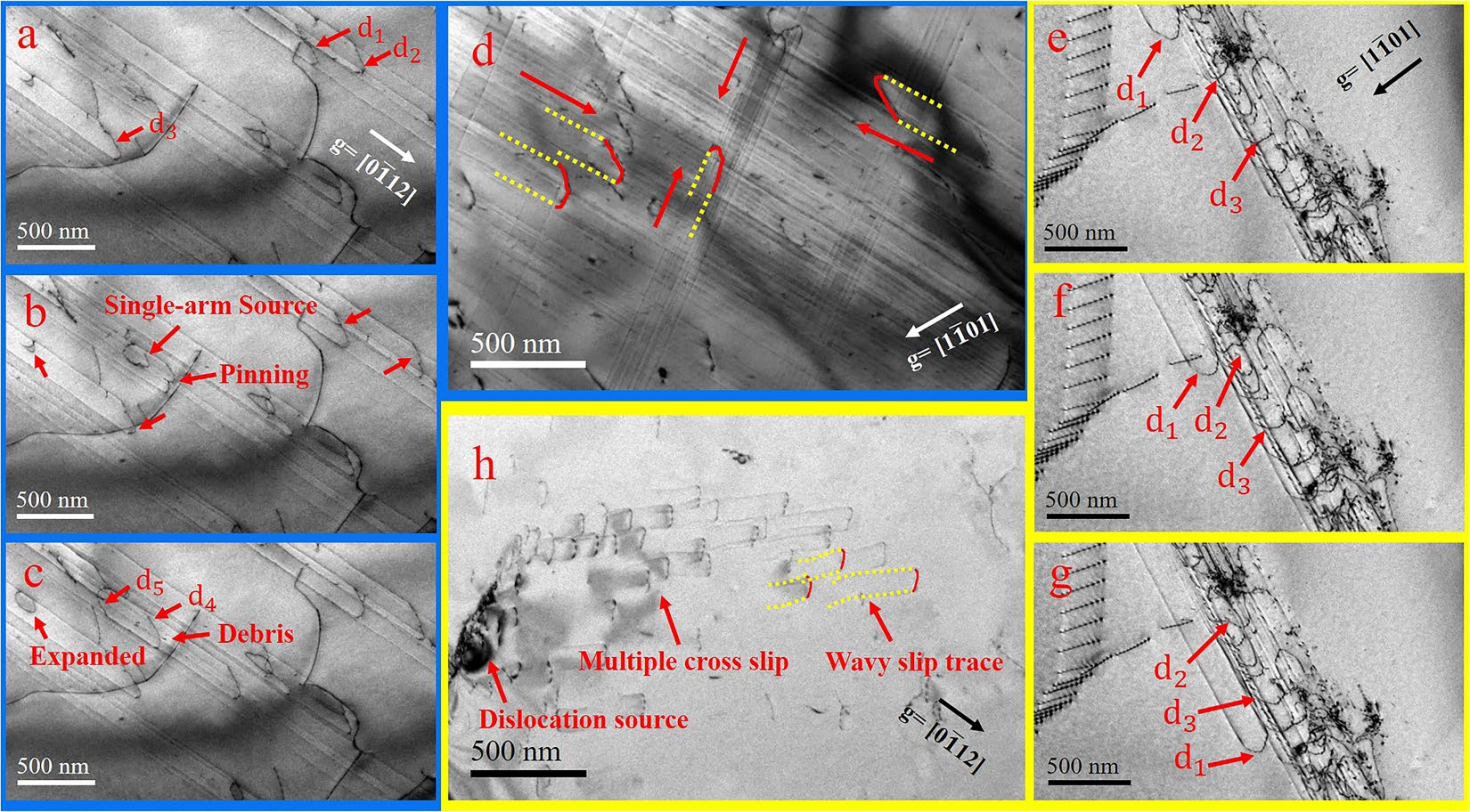
Screw dislocation movements in Ti (**a**–**c**) with g = $[0\bar{1}12]$ and (**d**) with g = $[1\bar{1}01]$ at room temperature. Screw dislocation movements in Ti-5at%Al (**e**–**g**) with g = $[1\bar{1}01]$ and (**h**) with g = $[0\bar{1}12]$ which are same as (**d**) and (**a**–**c**) specifically at room temperature.

The samples were further strained in TEM and a comparison of the defect activities of high-purity Ti and Ti-5at%Al were made at both room and liquid-nitrogen temperature. Figure 2(a–c) showed dislocation movements in high-purity Ti with view direction along g = $[0\bar{1}12]$ while Fig. 2(e–g) were dislocation movements in Ti-5at%Al perpendicular to g = $[1\bar{1}01]$ at room temperature. As shown in Fig. 2(a), as the external stress reached high enough, several slip systems (such as d_1_, d_2_ and d_3_) were activated at the same time, planar slips of screw dislocations occurred. The glide of dislocations sometimes met obstacles and could be temporarily pinned by them. However, in high-purity Ti, dislocation glide can overcome those barriers and continue to move with tiny debris left behind; the dislocation moved quickly and steadily in general. As external stress increases, dislocation multiplication occurred. As shown in Fig. 2(b,c), from a single-arm dislocation source, new dislocation d_4_ and d_5_ were generated and keep moving along planar plane towards converse orientation in succession. Similarly, as the red arrow marked in the left edge of the Fig. 2(b), another single-arm dislocation source formed in the type of dislocation loop. Then, after expanding in Fig. 2(c), from this loop, new dislocations emerged and moved in the same way as d_4_ and d_5_. Besides, planar slips can also occur along different directions as Fig. 2(d) showed at the same time. In contrast, at room temperature in Ti-5at%Al, as Fig. 2(e–g) showed, the way of dislocation movements did hardly change, where screw dislocation mainly moved by planar slip on prismatic planes along $[\bar{1}2\bar{1}0]$ direction. However, the glide of dislocations was not smooth compared to the similar planar slip in high-purity Ti. For instance, in Fig. 2(e), after the sluggish glide of d_1,_ d_2_ and d_3_ lipped along the same planar slip band but with sluggish motion as well. The details of dislocation movements can be found in the Supplementary Video. However, in Ti-5at%Al, cross-slip of dislocations were casually observed. As shown in Fig. 2(h), some screw dislocations did cross-slip by which they changed slip planes and directions quite frequently so that their slip trace were wavy at ten nanometer scale, which is at odds with the phenomenon of high-purity Ti that had been observed in the room temperature. In sum, the addition of 5 atomic percentage of Al indeed made dislocation glide more difficult. It is thought that although the motion of edge dislocations in both high-purity Ti and Ti-5at%Al did not prevail, the segregations of Al in Ti-5at%Al may generate strong barriers, promoting cross-slips.

**Figure 3:**
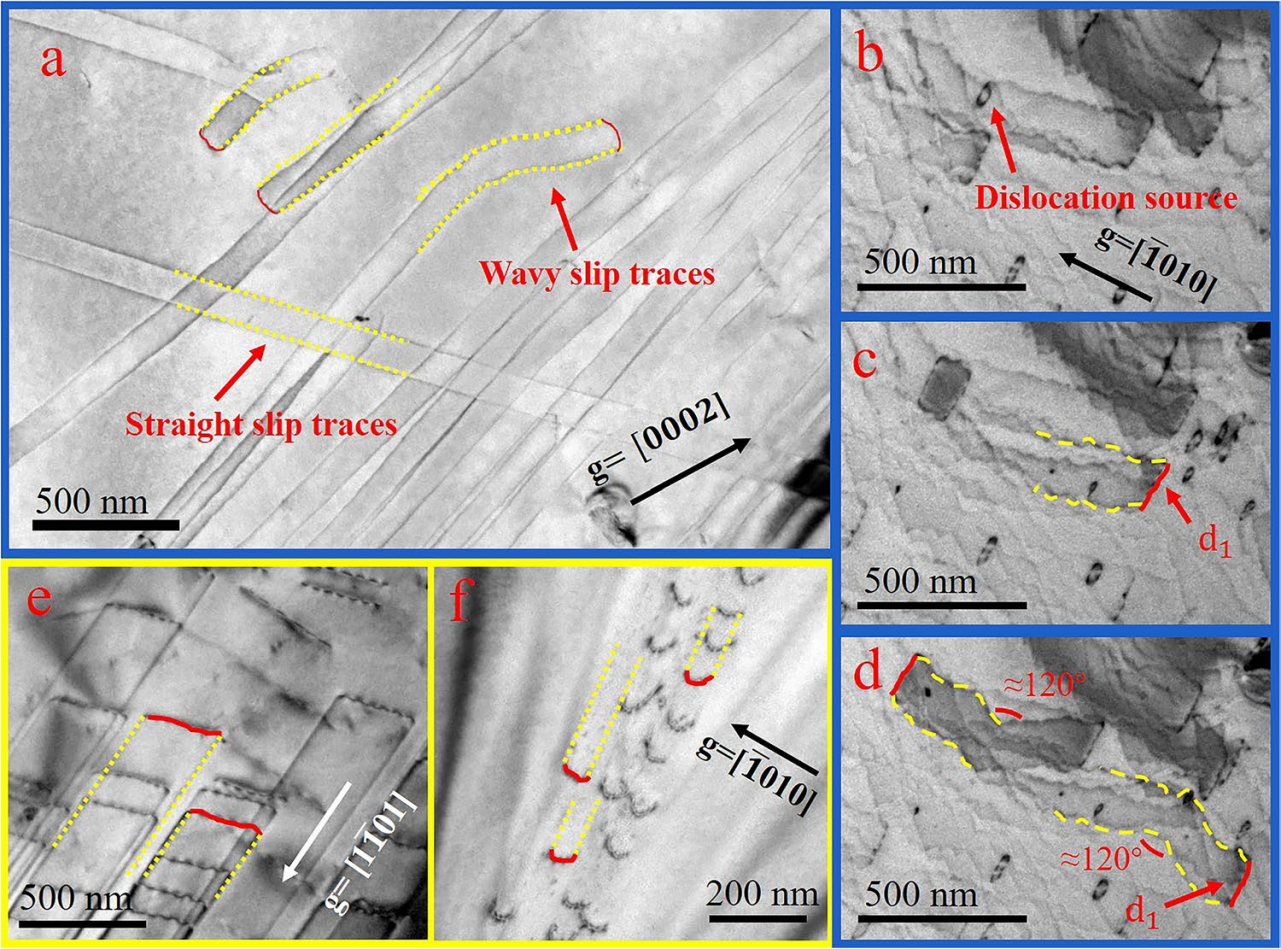
Screw dislocation movements in Ti (**a**) with g =$[0002]$ and (**b**–**d**) with g =$[\bar{1}010]$ at liquid-nitrogen temperature. Screw dislocation movements in Ti-5at%Al (**e**) with g =$[1\bar{1}01]$and (**f**) with the same g =$[\bar{1}010]$ as (**b**–**d**) at liquid-nitrogen temperature.

As it was discussed in the first part, the dislocation activities would be affected by the decrease of temperature due to the change of lattice vibration. Therefore, the dislocation motion would be harder and materials become stronger. In this study, as we performed *in-situ* TEM straining tests at liquid-nitrogen temperature, it was surprisingly found a contrary result. Figure 3(a–d) displayed the dislocation movements of Ti at liquid-nitrogen temperature under strain. Our systematic dislocation analysis demonstrated that the type of mobile dislocations was <a> type and the slip planes were on prismatic planes, which were the same compared to the room temperature characters. However, at low temperature, it was broadly observed that screw dislocations in high-purity Ti no longer slipped only through planar slip way, they displayed “snake-like” slip traces which indicated that frequent cross slip occurred during their glide, a phenomenon similar to that casually observed in the room temperature deformation of Ti-5at%Al.

For instance, as shown in Fig. 3(c,d), dislocation d_1_, which originally glided on a prismatic plane $(10\bar{1}0)$, did a large number of local cross-slips with only ten nanometer distance between each cross-slip event. At certain point, d_1_ cross-slipped to another prismatic plane $(\bar{1}010)$, and started to local cross-slip based on this new prismatic plane. The dislocations freshly generated from the dislocation sources also glided by local cross-slips. Eventually, the dislocation interactions were much heavily since the possibility for dislocations to encounter with others significantly increases due to massive cross-slips. In contrast, in Ti-5at%Al, no cross slips were observed, and screw dislocations slipped along a prismatic plane and formed planer slip as Fig. 3(e,f) displayed. Both in Ti and Ti-5at%Al at low temperature, dislocation movements cannot keep steadily and smoothly but become jerky, meanwhile multi-slip rarely happened. More details of dislocation movements can be found at the Supplementary Video. Nevertheless, the dislocation motion at liquid-nitrogen temperature was apparently more difficult in both high-purity Ti and Ti-5at%Al than that at room temperature. However, comparing these two materials, the massive cross-slip in high-purity Ti indicated that the motion of dislocations in high-purity Ti is even harder than that in Ti-5at%Al at low temperature, which was thought to be unlikely before.

**Figure 4:**
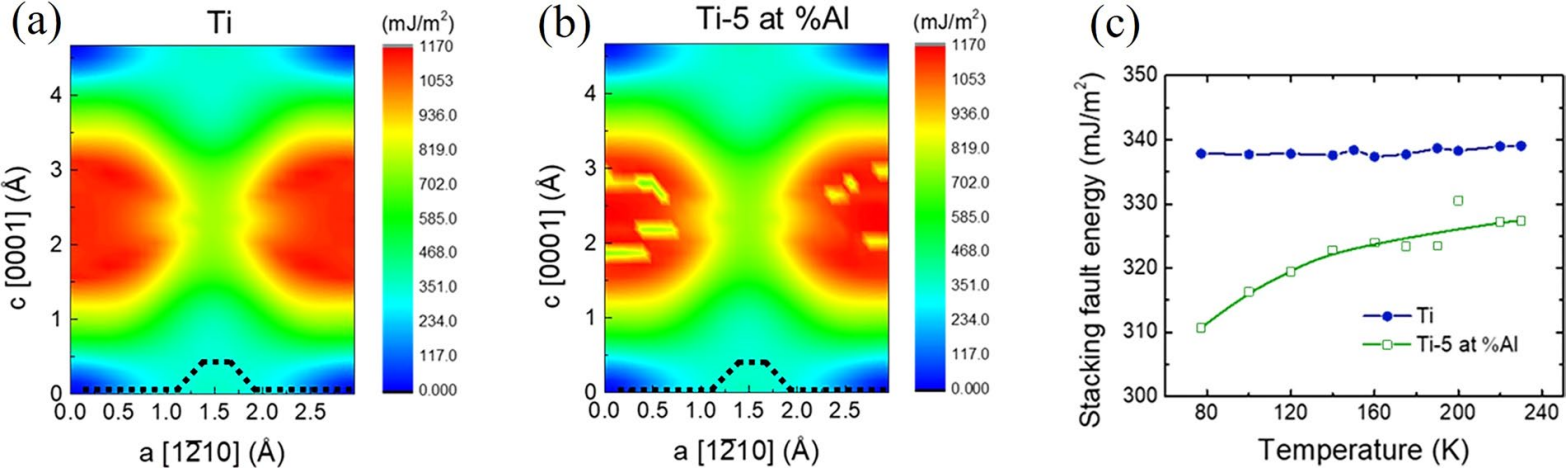
General stacking fault energy γ-surface in prismatic plane {10$\bar{1}$0} for (**a**) high-purity Ti and (**b**) Ti-5at%Al. The black dashed lines identify minimum energy path when sliding along a ([1$\bar{2}$10]) direction. (**c**) The variation of stacking fault energy with temperatures ranging from 77 K to 230 K for high-purity Ti and Ti-5at%Al.

To study the influence of Al on the dislocation plasticity in α-Ti, we further calculated the stacking fault energy which could be affected by the concentration of aluminium, meanwhile affect metal deformation behaviour^17^ by using the embedded atom method (EAM)^26^. We first calculated the γ-surface in prismatic {1$\bar{1}$00} plane for both high-purity Ti and Ti-5at%Al, as shown in Fig. 4(a,b), respectively. The minimum energy path is identified along the dashed black lines. For high-purity Ti, there is a saddle position when sliding along a-[1$\bar{2}$10] direction. The energy minimum is not located right at position of 0.5a, but with a small shift at the position of [0.5a, 0.1c]. The structure at the saddle position forms a stacking fault in prismatic {1$\bar{1}$00} plane. Adding Al could alter the energy locally in γ-surface, but does not change the minimum energy path and position of stable stacking fault. We further investigate the temperature effect on stacking fault energy. We take the structure at saddle point and optimize the structure by using Monte Carlo method^27^ at the temperatures ranging from 77  K to 230  K. Figure 4(c) shows the variation of stacking fault energy as the function of temperature for high-purity Ti and Ti-5at%Al. The temperature takes a minor effect on stacking fault energy in high-purity Ti, which stays around 310 mJ/m^2^. However, it takes a noticeable effect on Ti-Al system, i.e., the stacking fault energy drops substantially as the temperature decreases while the SFE of Ti-5at%Al is always lower than pure Ti. Besides, according to the geometric phase analysis (GPA) in Ti and Ti-5at%Al, the difference of lattice strain between two materials could not be the factor transforming the dislocation behaviours. The details of the calculation and GPA can be found from the Supplementary Materials.

Through the results of experiments and calculation, dislocation behaviours can be explained. At room temperature, the segregation of Al atoms induces obstacles restraining the dislocation slip and decreases the SFE slightly but the induced obstacles have more effects resulting in the tendency of cross slips like both wavy slips and planar slips occurred in Ti-5at%Al at room temperature. At liquid-nitrogen temperature, influenced by the sharp reduction of SFE in Ti-5at%Al, the SFE dominated the dislocation movements rather than the lattice friction and obstacles from lower temperature and segregation of Al, respectively, leading to planar slips. However, the SFE keeps stable while lattice friction increases with temperature decreasing which is consistent with cross slips in Ti at liquid-nitrogen temperature.

In high-purity Ti, early studies indicated a locking-unlocking mechanism governing dislocation plasticity at low temperature^28,29^. The dislocation spreads in pyramidal plane but encounters a high lattice friction when gliding in pyramidal planes. It could travel a relatively long distance through cross-slip in prismatic planes. Our experimental results show the similar behaviour that many cross-slip events are observed at lower temperature. The movement of screw dislocation is completed by a kink-pair mechanism. As the temperature increases to room temperature, the thermally activated kink nucleation becomes much easier. The glide of dislocation turns to be smooth and continuous, consequently the wavy slip is replaced by planar slip.

To sum, combining the experimental and simulation results, it is revealed that the influence of alloying elements on deformation mechanisms of titanium is highly related to temperature due to the alloying induced change in stacking-fault energy (SFE). While segregation of Al induces strong obstacles, promoting cross-slips at room temperature, the effect of Al on reducing stacking-fault energy (SFE) as decreasing temperature is significant. Consequently, the lower SFE in Ti-5at%Al results in ordinary planar dislocation slip while massive dislocation cross-slips occurred in Ti at liquid-nitrogen temperature. Such temperature dependence of alloying effect is worth of special attention since the application of alloys usually faces various environments.

## Methods

The TEM samples of high-purity Ti were prepared using twin-jet polishing in a methanol solution containing 5 vol.% perchloric acid at −30 °C while samples of Ti-5Al% were prepared by using a methanol solution containing 10 vol.% perchloric acid and 15 vol.% n-butanol. The atomic structure, specifically the crystal and chemical arrangements of Ti and Al atoms at edge and screw dislocation core were investigated by using high angle annular dark field scanning transmission electron microscope (HAADF-STEM) (FEI Titan) equipped with a large angle Chemi-STEM energy dispersive X-ray spectroscopy (EDS). *In-situ* straining experiments were processed by using tensile holder with samples glued in the substrate and the details of experiments method were demonstrated in previous literature^25^. The dynamic dislocation activities and other defects activities were observed by applying *in-situ* TEM straining testes operated at both room and liquid-nitrogen temperature in a FEI Tecnai G2 F20 TEM operating at 200 kV.

## Supplementary information

Supporting Information.

Supporting Information 2.

Supporting Information 3.

Supporting Information 4.

Supporting Information 5.

Supporting Information 6.
