# Supplementary figures and images for: Temperature Effect on Stacking Fault Energy and Deformation Mechanisms in Titanium and Titanium-aluminium Alloy
Source: Sci Rep. 2020 Feb 20;10:3086. doi: 10.1038/s41598-020-60013-6 (PMC7033204; doi:10.1038/s41598-020-60013-6)

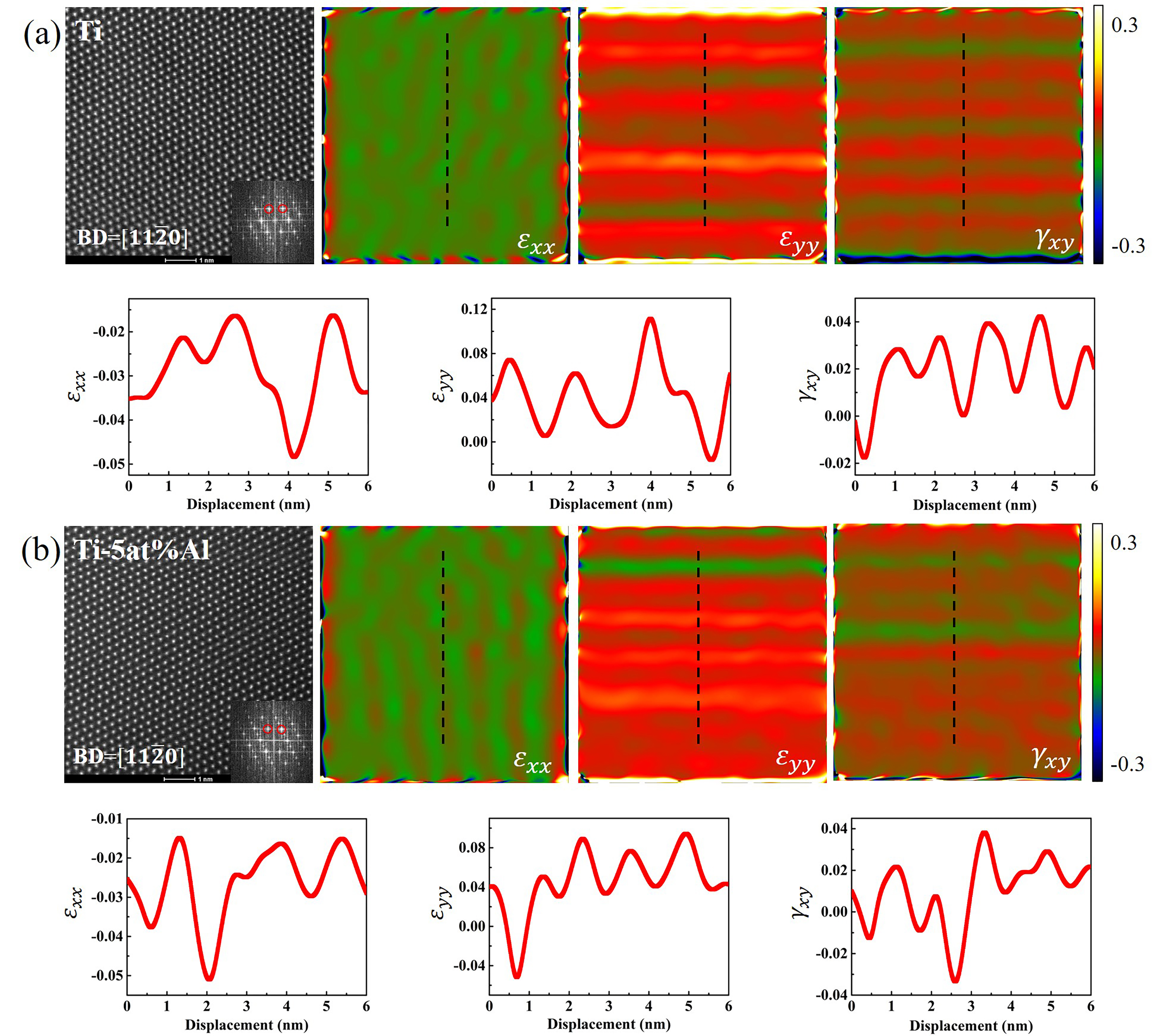

Supplement: Supplementary file 5 — Supporting Information 5. [file 41598_2020_60013_MOESM5_ESM.jpg]
