# Supplementary material for: Temperature Effect on Stacking Fault Energy and Deformation Mechanisms in Titanium and Titanium-aluminium Alloy
Source: Sci Rep. 2020 Feb 20;10:3086. doi: 10.1038/s41598-020-60013-6 (PMC7033204; doi:10.1038/s41598-020-60013-6)
Supplement: Supplementary file 6 — Supporting Information 6. [file 41598_2020_60013_MOESM6_ESM.docx]

**Supplementary Material**

Temperature Effect on Stacking Fault Energy and Deformation Mechanisms in Titanium and Titanium-aluminium Alloy

Beikai Zhao^1^, Peng Huang^1^, Libo Zhang^2^, Suzhi Li^2^, Ze Zhang^1^ and Qian Yu^1^*

^1^Center of Electron Microscopy and State Key Laboratory of Silicon Materials, School of Materials Science and Engineering, Zhejiang University, Hangzhou 310027, China; ^2^State Key Laboratory for Mechanical Behavior of Materials, Xi'an Jiaotong University, Xi'an 710049, China

*Corresponding author. Email address: yu_qian@zju.edu.cn.

**1. Movies**

Movie 1: In-situ transmission electron microscope (TEM) straining test of high-purity Ti at room temperature. The test showed dislocations glided quickly and steadily in the way of planar slip.

Movie 2: In-situ TEM straining test of Ti-5at%Al at room temperature. Dislocation in Ti-5at%Al moved sluggishly.

Movie 3: In-situ TEM straining test of high-purity Ti at liquid-Nitrogen temperature. Massive cross slips of dislocations occurred at low temperature and displayed “snake-like” slip traces.

Movie 4: In-situ TEM straining test of Ti-5at%Al at liquid-Nitrogen temperature. Dislocation movements cannot keep steadily and become jerky at this temperature.

**2. Details of the embedded atom method**

We use the embedded atom method (EAM) potential developed by Zope and Mishin^1^ to describe the interatomic interactions of Ti-Al system. This potential gives a proper fit to the basic properties of high-purity Ti, pure Al and Ti-Al. To account for the experimental observations on dislocation behaviour, we calculated the generalized stacking fault energy (GSFE) γ-surface in prismatic plane (1$\bar{1}$00). The γ-surface shows the energy landscape about the excess energy when one block of atoms are displaced by a rigid in-plane shift with respect to the other block. For calculation of γ-surface, we constructed a simulation box with orientations of x-[11$\bar{2}$0], y-[1$\bar{1}$00], z-[0001]. The size is around 70.8nm × 30.7nm × 56.1nm. Periodic boundary conditions were applied along x and z directions, while the free boundary condition was imposed along y direction. The energy landscape was then mapped out by shifting the block relative to the other, as a function of in-plane displacement along crystallographic a and c directions respectively, followed by the displacement relaxation in the out-of-plane direction using the conjugate gradient method. The minimum energy path with slipping along a direction and corresponding the stable stacking fault energy were then obtained in the map. To probe the effect of alloying element Al on the stacking fault energy, we introduced Al atoms with the atomic ratio of 5% into Ti randomly and use the same procedure to study the effect of Al on the energy landscape. To finally study the influence of temperature on the stacking fault energies, we relaxed the structure containing stable stacking fault at temperatures ranging from 77 K to 230 K for both high-purity Ti and Ti-5at%Al and then used Monte Carlo method^2^ to fully optimize the system. In specific, the swaps of Al and Ti is made with a probability min {1, exp(-∆E/k_B_T)}, where ∆E is energy difference before and after swap, k_B_ is the Boltzmann constant and T is temperature. The implement of MC simulations can optimize the configuration to an energy-favored state. For instance, the stacking fault energy decreases about 10.2% for Ti-5at%Al after MC optimization at liquid nitrogen temperature. All the simulations are carried out by using LAMMPS code^3^.

**3. Geometric phase analysis in Ti and Ti-5at%Al**


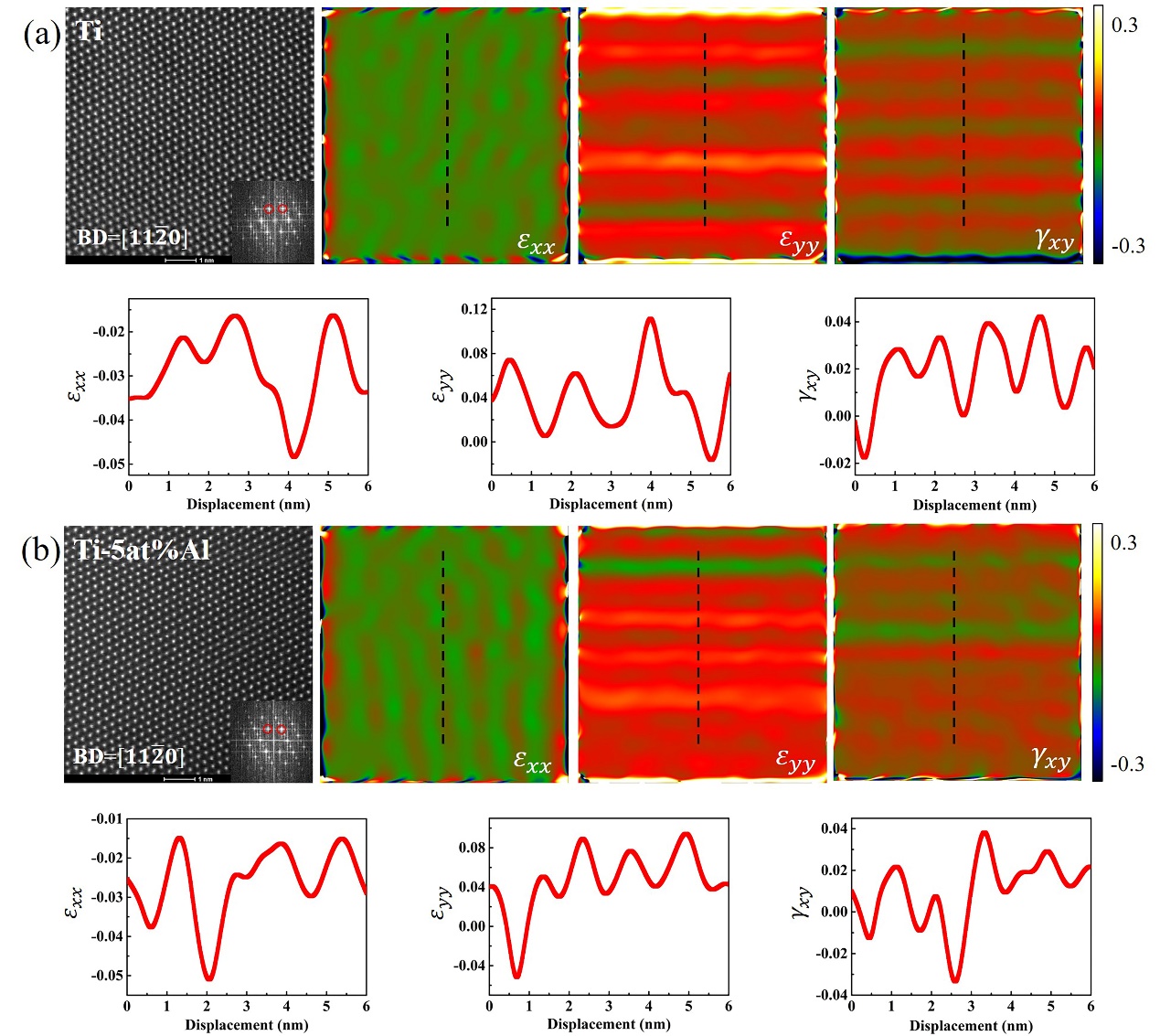


Figure S1. HAADF-STEM images and geometric phase analysis of $\varepsilon_{xx}$,$\varepsilon_{yy}$,$\gamma_{xy}$ with the related profiles in (a) Ti and (b) Ti-5at%Al.

We used geometric phase analysis (GPA) developed by MJ. Hÿtch, E. Snoeck and R. Kilaas^4^ to analyse the distribution of lattice strain through HAADF-STEM images. In Fig S1 (a) and (b), the zone axis of two samples are both $[11\bar{2}0]$ and identical diffraction points had been chosen for GPA. The horizontal normal strain ($\varepsilon_{xx}$), vertical normal strain ($\varepsilon_{yy}$) and shear strain ($\gamma_{xy}$) were displayed in Fig S1 (a) and (b) showing no highlights of strain in both of the materials in complete lattices. And no distinct difference in fluctuations of lattice strain between two materials can be observed in Fig S1 (a) and (b). So, the lattice strain should not be the reason for the different dislocation behaviours in the two materials.

**References**

1. Zope, R. R. & Mishin, Y. Interatomic potentials for atomistic simulations of the Ti-Al system. *Physical Review B* **68**, 024102 (2003).

2. Metropolis, N., Rosenbluth, A. W., Rosenbluth, M. N., Teller, A. H. & Teller, E. Equation of state calculations by fast computing machines. *The journal of chemical physics* **21**, 1087-1092 (1953).

3. Plimpton, S. Fast parallel algorithms for short-range molecular dynamics. *Journal of computational physics* **117**, 1-19 (1995).

4. Hÿtch, M., Snoeck, E. & Kilaas, R. Quantitative measurement of displacement and strain fields from HREM micrographs. *Ultramicroscopy* **74**, 131-146 (1998).
